# Prospective Association between Total and Trimester-Specific Gestational Weight Gain Rate and Physical Growth Status in Children within 24 Months after Birth

**DOI:** 10.3390/nu15214523

**Published:** 2023-10-25

**Authors:** Ke Wang, Bingzi Shang, Peiqi Ye, Qian Wei, Yunhui Zhang, Huijing Shi

**Affiliations:** 1 Key Laboratory of Public Health Safety, Ministry of Education, Department of Maternal, Child and Adolescent Health, School of Public Health, Fudan University, Yixueyuan Road, 138, Shanghai 200032, China; 20111020044@fudan.edu.cn (K.W.); 22211020096@m.fudan.edu.cn (B.S.); 20111020045@fudan.edu.cn (P.Y.); 18111020020@fudan.edu.cn (Q.W.); 2 Key Laboratory of Public Health Safety, Ministry of Education, Department of Environmental Health, School of Public Health, Fudan University, Yixueyuan Road, 138, Shanghai 200032, China; yhzhang@shmu.edu.cn

**Keywords:** gestation weight gain rate, trimester-specific, physical growth, longitudinal study

## Abstract

In this study, our aim was to investigate the potential correlation between the mother’s total gestational weight gain (GWG) rate and the trimester-specific GWG rate (GWGR) with the physical development status of the child within 24 months of age. We utilized linear regression models and linear mixed effects models to explore both time point and longitudinal relationships between GWGR and children’s anthropometric outcome z-scores at 0, 1, 2, 4, 6, 9, 12, 18, and 24 months. To examine the critical exposure windows, we employed multiple informant models. We also conducted a stratified analysis considering pre-pregnancy BMI and the gender of the children. Our findings revealed notable positive associations between total GWGR and z-scores for body mass index for age (BMIZ), head circumference for age (HCZ), weight for age (WAZ), length for age (LAZ), and weight for length (WHZ) across different trimesters of pregnancy (pint < 0.05). The GWGR during the first two trimesters mainly influenced the relationship between total GWGR and BMIZ, WAZ, and LAZ, while the GWGR during the first trimester had a significant impact on the correlation with HCZ (0.206, 95% CI 0.090 to 0.322). Notably, the associations of GWGR and children’s BMIZ were pronounced in male children and pre-pregnancy normal-weight women. In conclusion, our study findings indicated that a higher GWGR during each trimester was associated with greater physical growth during the first 24 months of life, especially GWGR in the first and second trimesters.

## 1. Introduction

Growth assessment and height and weight determination remain paramount in assessing public health and economic well-being, particularly in developing nations and countries undergoing transitions [1,2]. Physical growth status correlates directly to nutritional status and can reflect the health outcomes of children, encompassing concerns such as obesity, underweight, and chronic health conditions [3,4]. While the frequency of underweight persons has declined over the past 40 years, the incidence of obesity has grown everywhere [5,6]. This escalating trend in children’s body mass index (BMI) poses a global challenge, particularly in Asia [5,7]. Previous studies [8,9,10] have shown a positive correlation between excessive GWG (eGWG) and a larger birth size at term. Furthermore, eGWG has been associated with an augmented head circumference [11,12] and higher BMI throughout the lifespan [13,14,15,16,17,18,19,20,21,22,23]. The true direction and severity of the association between maternal weight gain and offspring BMI in the first two years of life is still mostly unclear [22], and a small amount of literature has studied this correlation in this time period [24,25], a sensitive period that increases the likelihood of lifelong health issues [26,27,28].

Furthermore, the weight gained during distinct intervals of pregnancy exhibits varied impacts on the physical growth status of offspring [29]. There is no consensus on the results of the time-sensitive period of the effect of GWG on children’s physical development [30,31,32,33], with the bulk of data coming from studies conducted in Western nations [8,34]. So far, literature topics on factors affecting the physical growth status of offspring include the gender-specific effects of prenatal and postpartum exposure, such as such as pre-pregnancy weight [35], breastfeeding [36], and smoking during pregnancy [37,38]. However, few studies have comprehensively investigated the gender-specific effects of trimester-specific GWG on the physical growth status of offspring following birth. Identifying these associated features will enable the more precise identification of offspring at risk and help to determine the optimal timing for interventions to prevent physical growth issues.

Given the existing limitations, we initiated a prospective birth cohort research study in China to better understand the relationship between maternal GWGR and children’s physical development. Our primary hypothesis was that total GWGR would demonstrate a positive correlation with the physical growth status of children. Additionally, we anticipated that the associations between total GWGR and various indicators of children’s physical growth would be influenced by the GWGR during different trimesters. Furthermore, we postulated that the relationship between total GWGR or GWGR in specific trimesters and the physical growth status of children might vary based on the pre-pregnancy BMI of women or the sex of the children.

## 2. Materials and Methods

### 2.1. Study Samples

Participants were drawn from the Shanghai Maternal-Child Pairs Cohort (Shanghai MCPC), a prospective birth cohort that began in 2016. Women from the Pudong and Songjiang District maternity hospitals in Shanghai made up this cohort. Briefly, eligible women had to be at least 20 years of age, with a minimum one-year residency in Shanghai, intending to undergo prenatal care and delivery at either of these two hospitals, able to complete Chinese questionnaires without difficulty, and willing to remain in Shanghai for more than three years post-delivery (details on enrollment requirements and processes have already been provided) [39]. In total, 6714 people were eventually enrolled in the cohort (the gestational age at enrollment was 12.14 ± 4.92 weeks). The population demographics during the period of enrollment are given in Supplementary Table S1. To gather comprehensive data, we utilized administered questionnaires and medical records to collect socio-demographic and clinical information from pregnant women during their initial prenatal visit. Additionally, we conducted research visits at the Community Health Center at the ages of 1, 2, 4, 6, 9, 12, 18, and 24 months to obtain detailed anthropometric measurements. Furthermore, we collected behavioral and environmental information from the mothers through questionnaires at the ages of 2, 4, 6, and 12 months.

**Figure 1:**
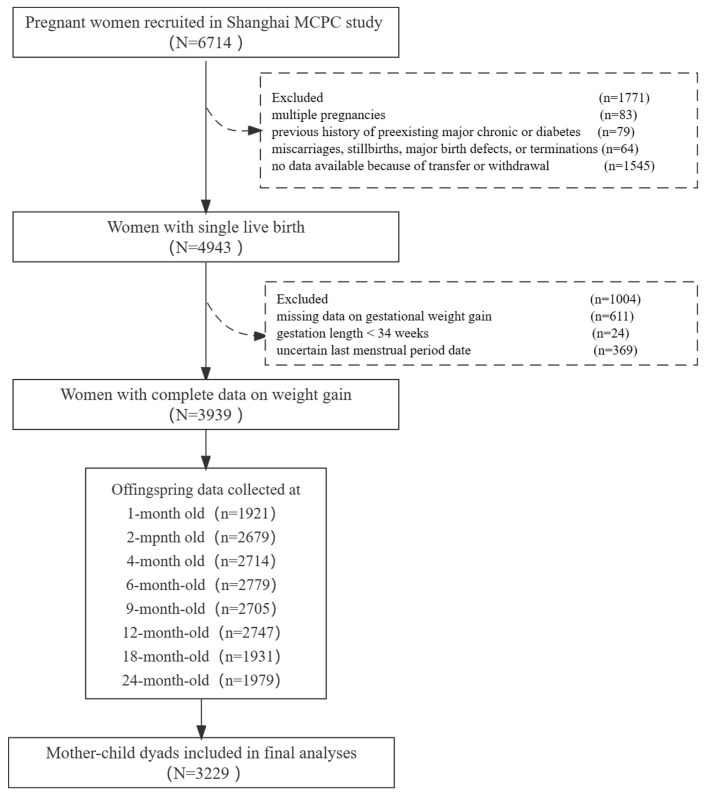
Flow diagram for subject selection.

According to the exclusion process of mother–child dyads with multiple pregnancies (n = 83), mother–child dyads with serious chronic diseases (chronic hypertension, diabetes, etc.) (n = 79), cases involving miscarriages, stillbirths, major birth defects, or terminations (n = 64), as well as instances where there were no available data due to transfer or withdrawal (n = 1545), a final sample size of 4943 pregnant women with single live births remained. Among these women, 3939 individuals had comprehensive data on pre-pregnancy weight, delivery weight, and weight records at each prenatal examination. Out of the 3939 pregnant women, 3229 actively participated in at least one follow-up visit (Figure 1). The average gestational age at enrollment for these 3229 pregnant women was 12.46 ± 4.31 weeks. We conducted a comparison of the characteristics between the included and excluded populations, and no significant differences were found (Table S2). The response rates for the follow-up visits at the 1, 2, 4, 6, 9, 12, 18, and 24 month follow-up were 48.8%, 68.0%, 68.9%, 70.6%, 68.7%, 69.7%, 49.0%, and 50.2%, respectively. The protocol for this research was approved by the Institutional Review Board at the School of Public Health at Fudan University in April 2016 (IRB number 2016-04-0587) and again in March 2020 (IRB number 2016-04-0587-EX). All participants provided written informed consent before enrollment and before each subsequent survey. At all times, participant rights were respected and ethical considerations were upheld.

### 2.2. Total and Trimester-Specific Gestational Weight Gain Rate

Pre-pregnancy weight was gathered through questionnaires or clinical records, while clinical records provided a median of 9 (range 3 to 19) repeated measurements of pregnancy weight per woman.

The GWG was calculated in this study by deducting the participant’s reported pre-pregnancy weight from the last clinically recorded weight before delivery. The disparity equaled all GWG. The GWGR was determined by dividing the total GWG by the total number of weeks of pregnancy.

Clinically relevant time points were utilized to evaluate GWG at different stages of pregnancy. First-trimester GWG was determined by subtracting the self-reported baseline weight from the weight on day 91 (13 weeks) of pregnancy [40]. By deducting the weight on day 91 (13 weeks) from the weight on day 196 (28 weeks) [24,41], we were able to calculate the GWG for the second trimester. Finally, the GWG for the third trimester was determined by subtracting the weight on day 196 (28 weeks) from the final weight recorded before delivery. We used linear interpolation with the two nearest weight data to approximate weights at day 91 and day 196 [24,40] when no data were available for actual weight measurements at those times. We calculated the GWGR for each trimester by dividing the weight gain by the total number of weeks in each trimester. We used 13 weeks for the first trimester, 28 weeks for the second, and the number of weeks from the end of the second trimester to delivery for the third.

### 2.3. Children’s Anthropometric Measures

The offspring’s weight and length were meticulously measured and recorded at birth, along with eight subsequent time points, specifically at ages 1, 2, 4, 6, 9, 12, 18, and 24 months. Additionally, their head circumference was measured and recorded at the same time points from age 1 to 24 months. All measurements and recordings were diligently performed by trained child healthcare doctors, ensuring accuracy and consistency throughout the study.

We transformed offspring z-scores of children’s length, weight, BMI, and head circumference into five indices using the World Health Organization’s recommendations for healthy child growth and development: weight-for-age z-score (WAZ), length-for-age z-score (LAZ), BMI-for-age z-score (BMIZ), weight-for-length z-score (WHZ), and head-circumference-for-age z-score (HCZ). This was performed using the WHO Anthro software (http://www.who.int/childgrowth/software/en/ (accessed on 1 January 2023)). The transforming function was the Z-scores of children’s anthropometric data (length, weight, BMI, and head circumference) = (actual measured value—median (M) of the reference population)/standard deviation (SD) of the reference population.

### 2.4. Study Covariates

We collected the mother’s socio-demographic data (i.e., education, annual family income) and father’s socio-demographic data (i.e., height, weight, age) via questionnaires at the baseline inclusion of the study population. We also recorded the prevalence of maternal alcohol use before pregnancy and passive smoking throughout pregnancy. Due to the small sample size, we did not include smoking status (6/3229) and alcohol use (1/3229) during pregnancy as independent variables in our analysis. We also collected pregnancy complications (any one of clinically diagnosed gestational hypertension, gestational diabetes, and preeclampsia was identified as a pregnancy complication), maternal age at delivery, maternal parity (primiparous or non-primiparous), delivery mode (natural birth or caesarean section), and children’s sex (male or female) from clinical and birth records. During offspring follow-up, we collected information on complementary food additions, breastfeeding duration, and the mode of children’s feeding, which was categorized as breast milk, a combination of breast milk and formula, and just formula [24,42]. For the purpose of calculating maternal energy intake in the third trimester, we employed the food exchange unit based on data from the semi-quantitative Food Frequency Questionnaire [43]. Based on a gestational age birthweight percentile, children were classified into three groups, small for gestational age (SGA), appropriate for gestational age (AGA), and large for gestational age (LGA), based on their birth conditions [44]. The Chinese version of the PSQI, which has been validated and shown to be useful in measuring sleep quality in pregnant women [45], was used to conduct this study’s assessment of pregnant women’s sleep quality. Pregnant women’s activity levels were scored on the International Physical Activity Questionnaire (IPAQ) and categorized as low, medium, or high depending on the findings [46]. The Center for Epidemiologic Studies Depression (CES-D) [47,48] was used to evaluate mother depression, and the Self-Rating Anxiety Scale (SAS) [49,50] was used to measure maternal anxiety. These questionnaires assessed the mother’s emotional well-being during her pregnancy. The WGOC’s guidelines were used to classify the parents’ BMIs before conception based on their respective BMI categories.

### 2.5. Statistical Analysis

We divided mother–child pairs into four categories based on GWGR quartiles. Using ANOVA or chi-square testing, we compared variations in exposure and covariate features among GWGR quartiles.

The associations of total GWGR with the z-scores of children’s length, weight, BMI, and head circumference at each time point (at 0, 1, 2, 4, 6, 9, 12, 18, 24 months) were estimated by multivariable linear regression models. A multiple informant model was used to estimate the association between trimester-specific GWGR and children’s anthropometric outcome z-scores. Linear mixed effects (LME) models were used to examine the longitudinal associations of total and trimester-specific GWGR with offspring z-scores of weight, length, BMI, and head circumference from birth to 24 months, as this study involved repeated measurements of the physical data at eight time points of the same observation object. The cubic goodness of fit of the model was the best, and we achieved this by using the maximum likelihood estimation technique. We also conducted a stratified analysis considering pre-pregnancy BMI and the gender of the children.

For sensitivity analyses, we excluded women with gestational diabetes mellitus in the LME analysis, which can affect both weight gain [51] and the physical growth status of offspring [52,53]. The identification of confounders in the main analysis relied on previous studies. Two-tailed tests were performed, and results with a *p*-value less than 0.05 were deemed significant. The covariates had missing values ranging from 0.47% (maternal age) to 10.90% (maternal sleep quality). To handle the missing values, we employed multiple imputation using chained equations (MICE) and generated 20 imputed datasets. SAS 9.4, R version 4.1.2, and SPSS Statistics 26 were used for all of the statistical analyses.

## 3. Results

### 3.1. Participants’ Characteristics

When performing a comparison between women belonging to the lowest quartile of GWGR and those belonging to the top quartile, several features were observed in the latter group. The observed characteristics included a younger demographic, with a mean age of 28.25 ± 4.07 years, in contrast to the prior group’s mean age of 28.54 ± 4.12 years. Furthermore, it was observed that a higher percentage of primiparous women, comprising 61.21% of the sample, were present in the uppermost quartile, while the lower quartile exhibited a proportion of 50.19%.

Simultaneously, the results shown in Table 1 demonstrate that women in the top quartile of GWGR displayed greater GWGR values in the initial trimester (0.27 ± 0.28 vs. 0.10 ± 0.23 kg/week), the second trimester (0.60 ± 0.19 vs. 0.36 ± 0.17 kg/week), and the third trimester (0.75± 0.29 vs. 0.38 ± 0.33 kg/week); lower prevalence of pre-pregnancy overweight/obesity (10.90% vs. 26.89%); and lower prevalence of gestational diabetes mellitus (6.44% vs. 19.58%).

### 3.2. Effects of Total and Trimester-Specific GWGR on Children’s Physical Growth

Table 2 shows that there was a positive relationship between the z-scores for the anthropometric outcomes and the total GWGR. To be more specific, it demonstrates that a greater level of total GWGR was associated with higher levels of BMIZ, HCZ, WAZ, LAZ, and WHZ across the board. Meanwhile, the relationships of GWGR with BMIZ, HCZ, WAZ, LAZ, and WHZ significantly differed for different trimesters of gestation (pint < 0.05).

Table 3 displays the results of a longitudinal study that focused on the impact of total or trimester-specific GWGR on the anthropometric z-scores of children within 2 years of birth. Positive associations were found between total GWGR and BMIZ, HCZ, LAZ, WAZ, and WHZ. Total GWGR was significantly correlated with BMIZ, with the second-trimester GWGR having the strongest effect (0.312, 95% CI 0.171 to 0.452), followed by the first-trimester GWGR (0.133, 95% CI 0.034 to 0.223). Similarly, the GWGR in the second trimester (0.339, 95% CI 0.196 to 0.482) and the GWGR in the first trimester (0.227, 95% CI 0.125 to 0.330) had the greatest impact on the association between total GWGR and WAZ. The first-trimester GWGR (0.253, 95% CI 0.136 to 0.370) and second-trimester GWGR (0.192, 95% CI 0.028 to 0.356) were shown to have the greatest impact on the association between LAZ and total GWGR. Furthermore, the GWGR in the first trimester had the greatest impact on the link between total GWGR and HCZ (0.206, 95% CI 0.090 to 0.322). In the sensitivity analysis, excluding women with gestational diabetes mellitus in the LME analysis, we found similar results of the association between total or trimester-specific GWGR and children’s anthropometric outcome z-scores (Table S3).

### 3.3. Association of Total and Trimester-Specific GWGR with Children’s BMIZ by Pre-Pregnancy BMI

**Figure 2:**
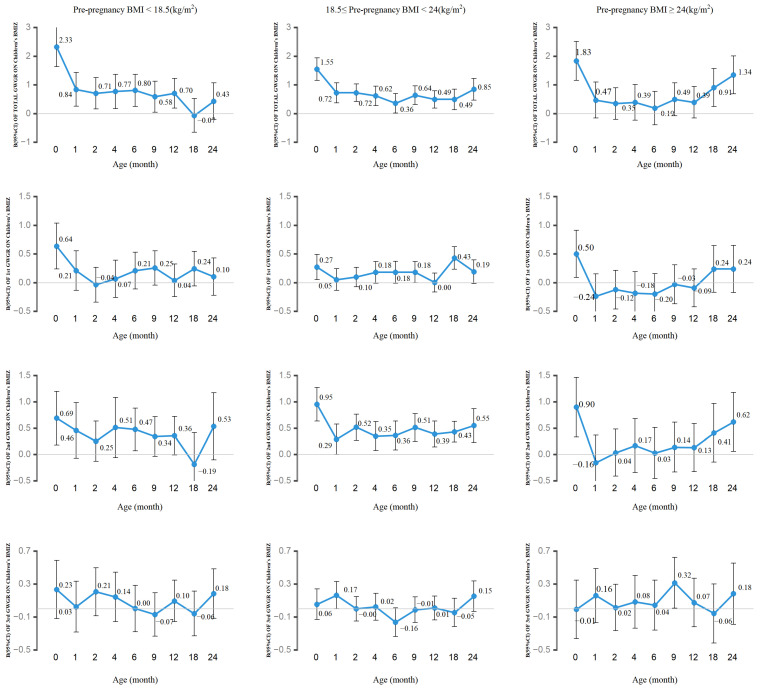
Forest plot of β with 95% CIs of BMIZ of 0-to-24-month-old offspring based on pre-pregnancy weight-specific total and trimester-specific GWGR. Adjusted for age at delivery, maternal pre-pregnancy BMI, education, income, physical activity, sleep quality, complications, parity, delivery mode, anxiety and depression in pregnancy, maternal passive smoking during pregnancy, maternal alcohol consumption before pregnancy, children’s sex, paternal age, paternal BMI, children’s age at anthropometric measurements, feeding mode, supplementary food addition. When analyzing the physical growth of children during postnatal follow-up, we adjusted for SGA, LGA, and AGA. The total GWGR models were further adjusted for energy intake in late pregnancy. The second GWGR models were further adjusted for 1st-trimester GWGR. The third GWGR models were further adjusted for 1st-trimester GWGR, 2nd-trimester GWGR, and energy intake in late pregnancy.

Using a pre-pregnancy BMI stratification (Figure 2), we found that total GWGR was positively associated with children’s BMIZ at all time points among normal-weight women. At all ages except 18 and 24 months, the total GWGR was positively associated with BMIZ in the offspring of underweight mothers. Only at ages 0, 18, and 24 did overweight or obese women exhibit a positive correlation between total GWGR and BMIZ. In addition, the GWGR in the second trimester was positively associated with the BMIZ of their progeny across all time periods among mothers of normal weight. There were no associations between second-trimester GWGR and children’s BMIZ at any follow-up nodes other than 0 and 24 months of age in overweight/obese women.

### 3.4. Association of Total and Trimester-Specific GWGR with Children’s BMIZ by Children’s Sex

**Figure 3:**
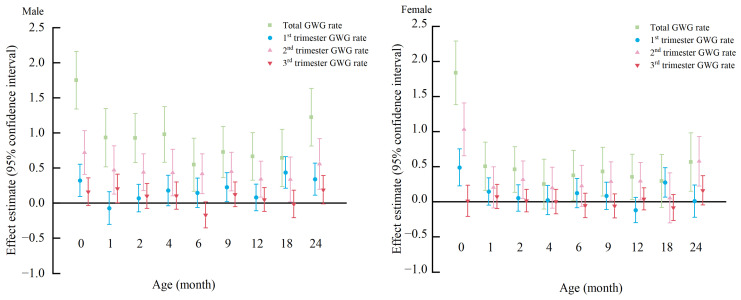
Forest plot of β with 95% CIs of BMIZ of 0-to-24-month-old offspring based on sex-specific total and trimester-specific GWGR. Adjusted for age at delivery, maternal pre-pregnancy BMI, education, income, physical activity, sleep quality, complications, parity, delivery mode, anxiety and depression in pregnancy, maternal passive smoking during pregnancy, maternal alcohol consumption before pregnancy, paternal age, paternal BMI, children’s age at anthropometric measurements, feeding mode, supplementary food addition. When analyzing the physical growth of children during postnatal follow-up, we adjusted for SGA, LGA, and AGA. The total GWGR models are further adjusted for energy intake in late pregnancy. The second GWGR models are further adjusted for 1st-trimester GWGR. The third GWGR models are further adjusted for 1st-trimester GWGR, 2nd-trimester GWGR, and energy intake in late pregnancy.

Using a sex-stratified analysis (Figure 3), we discovered that the total and trimester-specific GWGR were associated with children’s BMIZ in distinct ways for boys and girls. Male children showed a positive correlation between total GWGR and BMIZ across all ages. The total GWGR was favorably linked with girls’ BMIZ at all ages except 4 and 18 months. The second-trimester GWGR was positively associated with male children’s BMIZ at all time points; the positive relationship between the second-trimester GWGR and children’s BMIZ was reflected only at birth and 2, 9, 12, and 24 months old among female children.

## 4. Discussion

The present study was undertaken using a sample from Asia in order to investigate the association between maternal GWGR and offspring physical growth status within 2 years of birth. Children born to mothers with a greater total GWGR may have higher z-scores in terms of growth across all four dimensions (height, weight, and body mass index) before the age of two. The observed correlation between total GWGR and growth measurement z-scores (length, weight, and BMI) was mainly driven by the GWGR in mid-pregnancy and GWGR in early pregnancy. On the other hand, the observed association between total GWGR and growth measure z-scores of head circumference was primarily influenced by the GWGR in the first trimester. The study revealed a significant positive association between the total and trimester-specific GWGR and the BMIZ of children born to pregnant mothers with a normal weight. This association was especially pronounced in male offspring.

The children’s BMI increased significantly with increasing total GWGR, similar to previous studies [16,18,24]. One of the potential pathways is that the overnutrition in utero caused by GWG can expose the fetus to more fatty acids and glucose and lead to the excessive growth of offspring [54,55]. As was found in other studies [11,12], we also found a positive association between total GWGR and offspring head circumference. One possible mechanism is that a greater GWG can lead to a decrease in high-density lipoprotein cholesterol (HDL) levels in pregnant women [12], further positively affecting head circumference growth [56,57].

Compared to the total GWGR, the research results on the association of trimester-specific GWGR with children’s physical growth remains inconsistent. For example, in the Greek pregnancy cohort “Rhea” study, Karachaliou et al. identified a correlation between the GWGR in the first trimester of pregnancy and higher BMIZ at one year of age, as well as greater skinfold thickness at four years of age [58]. A positive association between GWGR during the first two trimesters and offspring BMIZ during the first six months of life was identified in the “Born in Shenyang Cohort Study” conducted by Hu et al. [24]. Nevertheless, the impact of the second trimester was not well examined in these studies since it was combined with either the third trimester or the first trimester. This work added to the existing body of knowledge by analyzing the correlation between GWGR during the first, second, and third trimesters of pregnancy and the subsequent physical development of the child. In the present study, we examined the critical exposure windows by using multiple informant models. Our research findings indicate that the GWGR in the second trimester may potentially serve as a critical period for interventions aimed at changing the body mass index of children. This finding aligns with previous research that has shown a correlation between the physiological activity of fetal adipose tissue, which begins around 14–16 weeks of gestation, and the BMI of children [25,30]. The onset of physiological activity in fetal adipose tissue typically occurs during the gestational period of 14–16 weeks [59]. Based on the results obtained from our research, it was seen that there existed a positive correlation between GWGR in the first trimester of pregnancy and the head circumference of children. However, this correlation was not evident in the subsequent trimesters. This phenomenon might perhaps be attributed to the rapid proliferation, migration, and organization of neurons during the first trimester of pregnancy [60,61]. In summary, these findings, combined with those of other investigators, confirm the importance of GWG in the first two trimesters, which is a highly sensitive period for offspring physical growth compared to the late pregnancy period.

The potential correlation between maternal GWGR throughout each trimester and child BMI in women with different BMI categories has yet to be investigated. The study found that there was a greater correlation between total GWGR and offspring BMI in women who were overweight before pregnancy [62]. Conversely, women who had a normal weight before pregnancy exhibited a more pronounced relationship between total GWGR and offspring BMI [24]. This research represented the first examination of the association between changes in GWGR during each trimester of pregnancy and children’s BMI over a diverse spectrum of pre-pregnancy body mass indices. The association between trimester-specific GWGR and the BMI of children showed a greater positive trend in mothers who had a normal pre-pregnancy weight. There are two possible reasons for this phenomenon. Firstly, the pre-pregnancy overweight/obese women who were included in this study intentionally controlled their weight gain, resulting in a lower GWGR. Secondly, the offspring of overweight/obese pregnant women before pregnancy are relatively heavier, resulting in a ceiling effect. Therefore, the target population for the prevention of excessive GWG and subsequently childhood obesity should be pregnant women with a normal pre-pregnancy weight, and they may obtain more advantages from interventions than those for underweight or overweight/obese pregnant women.

As was found in one study [24], we found that the correlation between total or trimester-specific GWGR and the BMI of offspring during the first two years of life was more prominent in male children. The existence of gender disparities may be attributed to several factors, such as societal conventions and inherent biological distinctions. From a biological perspective, it has been shown that female children have a higher likelihood of leptin production [63,64,65,66,67]. This hormone exerts its effects on the hypothalamus and other components of the central nervous system, resulting in a reduction in food consumption and an increase in energy expenditure [68,69]. In China, male individuals tend to receive more financial support from their families compared to females due to social contextual variables. This disparity in financial aid may potentially contribute to the issue of overfeeding and subsequent obesity [70].

We conducted a longitudinal study, which provided greater scope to distinguish correlations from causation. The cooperation with designated hospitals and community health service stations ensured the follow-up rate of the cohort population. By collecting weight values at multiple time points throughout the pregnancy and repeated-measures physical data of the offspring within 2 years after birth, we investigated the relationship between the total or trimester-special GWGR and the physical growth of the offspring. Furthermore, our study focused on mother–child dyads in China in order to mitigate the potential confounding influence of ethnic background. However, this study had some inherent limitations. The determination of the pre-pregnancy weight status relied on self-reported data, despite prior studies indicating a robust correlation between the self-reported and objectively measured pre-pregnancy weight [22,29]. Nevertheless, it is crucial to recognize the possible existence of memory bias within these accounts. In the present investigation, we included many possible confounding variables, including socio-demographic characteristics, maternity-related data, socioeconomic indicators, information pertaining to supplementary food intake, and the manner in which infants were fed. This measure was implemented in order to bolster the reliability of our results. Nevertheless, it is important to emphasize that our study did not include data on the individual energy intake of children, a factor that is generally recognized as having a substantial impact on their growth and development. Unfortunately, we only collected the dietary information during late pregnancy. Meanwhile, we regret that no further information was collected on the proportion of nutrients in breast milk and other foods in mixed feeding, resulting in the imprecise measurement of infant feeding. We also lacked data showing how markers such as the fat percentage and waist circumference caused by a maternal weight increase affected children’s development. The children enrolled in the Shanghai MCPC will undergo longitudinal monitoring until they reach the age of five, allowing for an evaluation of the enduring consequences of our research results. Moreover, we will conduct another analysis of this population by correlating the child growth findings with the fetal growth shown in u/s evaluations of this prospective study.

## 5. Conclusions

This research offers empirical evidence suggesting that a greater GWGR is positively related to offspring physical growth within 24 months; mainly, the first- and second-trimester GWGR affect the children’s weight and length, while the first-trimester GWGR affects the children’s head circumference. Additionally, the associations of GWGR and children’s BMIZ were pronounced in male children and pre-pregnancy normal-weight women. Further investigation is required to validate our findings and provide a strong biological foundation for the observed correlations. Nevertheless, our study offers vital insights into the specific duration and demographic group that should be targeted in order to successfully manage eGWG. It is advisable to prioritize the management of weight increase during the latter stages of pregnancy in current programs. However, it is essential to recognize the importance of managing weight gain throughout the early and mid-pregnancy phases as well. Additionally, it is crucial to give priority to the surveillance of weight growth in pregnant women who have a healthy weight before pregnancy. This should be done in conjunction with evaluating the weight status of male children, with the objective of reducing the probability of developing overweight or obesity in the future.

## Figures and Tables

**Table 1:** Characteristics of participants according to four groups by quartile of total GWGR (n = 3229).

Variables	Subjects, Mean ± SD	Quartile of Total GWGR, Mean ± SD or n (%)				*p*-Value
	or n (%)	Quartile 1	Quartile 2	Quartile 3	Quartile 4	
	n = 3229	n = 807	n = 806	n = 809	n = 807	
Maternal characteristics						
Age at delivery (years)	28.93 ± 4.09	28.54 ± 4.12	29.22 ± 4.11	28.69 ± 3.94	28.25 ± 4.07	<0.001
Education level						<0.001
Middle school and below	285 (8.82)	61 (7.56)	57 (7.07)	70 (8.65)	97 (12.02)	
High school or same level	410 (12.70)	106 (13.14)	91 (11.29)	100 (12.367)	113 (14.00)	
Junior college or same level	1081 (33.48)	251 (31.10)	254 (31.51)	281 (34.73)	295 (36.56)	
College and above	1453 (45.00)	389 (48.20)	404 (50.13)	358 (44.25)	302 (37.42)	
Annual family income						0.392
<¥100,000	771 (23.88)	183 (22.68)	185 (22.95)	199 (24.60)	204 (25.28)	
¥100,000–200,000	1551 (48.03)	385 (47.71)	388 (48.14)	380 (46.97)	398 (49.32)	
¥200,000–300,000	600 (18.58)	169 (20.94)	155 (19.23)	142 (17.55)	134 (16.60)	
≥¥300,000	307 (9.51)	70 (8.67)	78 (9.68)	88 (10.88)	71 (8.80)	
Second-hand smoking during pregnancy						0.307
Yes	781 (24.19)	196 (24.29)	180 (22.33)	213 (26.33)	192 (23.79)	
No	2448 (75.81)	611 (75.71)	626 (77.67)	596 (73.67)	615 (76.21)	
Alcohol before pregnancy	74 (2.29)	13 (1.61)	19 (2.36)	19 (2.35)	23 (2.85)	
Sleep quality during pregnancy						0.334
Good	1099 (34.04)	279 (34.57)	267 (33.13)	264 (32.63)	289 (35.81)	
Poor	2130 (65.96)	528 (65.43)	539 (66.88)	545 (67.37)	518 (64.19)	
Physical activity intensity						0.148
Low	1327 (41.10)	341 (42.26)	328 (40.69)	340 (42.03)	318 (39.41)	
Medium	1743 (53.98)	422 (52.29)	445 (55.21)	439 (54.26)	437 (54.15)	
High	159 (4.92)	44 (5.45)	33 (4.09)	30 (3.71)	520 (6.44)	
Depression during pregnancy	366 (11.33)	88 (10.90)	100 (12.41)	92 (11.37)	86 (10.66)	0.696
Anxiety during pregnancy	401 (12.42)	72 (8.92)	106 (13.15)	112 (13.84)	111 (13.75)	0.572
Parity						<0.001
Primiparous	1803 (55.84)	405 (50.19)	450 (55.83)	454 (56.12)	494 (61.21)	
Non-primiparous	1426 (44.16)	402 (49.81)	356 (44.17)	355 (43.88)	313 (38.79)	
Pre-pregnancy BMI (kg/m^2^)						<0.001
<18.5	543 (16.82)	105 (13.01)	149 (18.49)	142 (17.55)	147 (18.22)	
18.5–24	2170 (67.20)	485 (60.10)	550 (68.24)	563 (69.59)	572 (70.88)	
≥24	516 (15.98)	217 (26.89)	107 (13.27)	104 (12.86)	88 (10.90)	
Gestational diabetes	345 (10.68)	158 (19.58)	80 (9.93)	55 (6.80)	52 (6.44)	<0.001
1st trimester GWGR (kg/week)	0.17 ± 0.24	0.10 ± 0.23	0.15 ± 0.20	0.18 ± 0.2015	0.27 ± 0.28	<0.001
2nd trimester GWGR (kg/week)	0.48 ± 0.18	0.36 ± 0.17	0.45 ± 0.14	0.51 ± 0.12	0.60 ± 0.19	<0.001
3rd trimester GWGR (kg/week)	0.57 ± 0.31	0.38 ± 0.33	0.54 ± 0.26	0.62 ± 0.24	0.75± 0.29	<0.001
Paternal characteristics						
Age (years)	29.97 ± 4.62	30.67 ± 4.52	30.19 ± 4.67	29.77 ± 4.46	29.24 ± 4.71	<0.001
FBMI category (kg/m^2^)						0.486
<18.5	140 (4.34)	32 (3.97)	43 (5.33)	31 (3.83)	34 (4.21)	
18.5–24	1615 (50.02)	385 (47.71)	402 (49.88)	423 (52.29)	405 (50.19)	
≥24	1474 (45.64)	390 (48.32)	361 (44.79)	331 (43.88)	368 (45.60)	
Children’s characteristics						
Sex						0.391
Male	1651 (51.13)	400 (49.57)	409 (50.74)	409 (50.56)	433 (53.66)	
Female	1578 (48.87)	407 (50.43)	397 (49.26)	400 (49.44)	374 (46.34)	

**Table 2:** Multivariable linear regression (β (95% CI)) and multiple informant models (pint) of children’s anthropometric outcomes at 0, 1, 2, 4, 6, 9, 12, 18, 24 months according to total and trimester-specific GWGR (n = 3229).

Children Anthropometric Measures	Total GWGR ^c^	1st Trimester GWGR	2nd Trimester GWGR ^a^	3rd Trimester GWGR ^bc^	Pint ^d^
	(kg/week)	(kg/week)	(kg/week)	(kg/week)	
BMIZ					
0 month	1.838 (1.543, 2.133) ***	0.421 (0.253, 0.590) ***	0.929 (0.695, 1.162) ***	0.088 (−0.055, 0.231)	**<0.001**
1 month	0.714 (0.451, 0.977) ***	0.036 (−0.112, 0.184)	0.272 (0.051, 0.492) *	0.123 (−0.007, 0.254)	0.364
2 month	0.698 (0.459, 0.936) ***	0.065 (−0.071, 0.201)	0.374 (0.188, 0.560) ***	0.050 (−0.069, 0.169)	**0.042**
4 month	0.613 (0.347, 0.879) ***	0.105 (−0.046, 0.256)	0.320 (0.099, 0.541) **	0.053 (−0.078, 0.185)	0.297
6 month	0.450 (0.190, 0.710) ***	0.135 (−0.013, 0.284)	0.323 (0.120, 0.527) **	−0.114 (−0.242, 0.013)	**0.002**
9 month	0.602 (0.350, 0.854) ***	0.165 (0.022, 0.308) *	0.373 (0.178, 0.569) ***	0.039 (−0.083, 0.162)	0.069
12 month	0.533 (0.298, 0.767) ***	−0.009 (−0.141, 0.123)	0.310 (0.126, 0.495) **	0.050 (−0.066, 0.165)	**0.030**
18 month	0.486 (0.208, 0.764) ***	0.177 (−0.064, 0.418)	0.348 (0.193, 0.502) ***	−0.039 (−0.175, 0.096)	**0.003**
24 month	0.909 (0.617, 1.200) ***	0.173 (0.011, 0.335) *	0.542 (0.288, 0.795) ***	0.181 (0.036, 0.326) *	0.076
HCZ					
1 month	0.675 (0.371, 0.980) ***	0.182 (0.011, 0.353) *	0.138 (−0.117, 0.393)	0.016 (−0.135, 0.167)	0.575
2 month	0.480 (0.212, 0.748) ***	0.143 (−0.009, 0.295)	−0.009 (−0.217, 0.199)	−0.010 (−0.144, 0.123)	0.334
4 month	0.365 (0.090, 0.639) **	0.190 (0.035, 0.346) *	−0.128 (−0.356, 0.101)	−0.049 (−0.184, 0.086)	**0.033**
6 month	0.469 (0.204, 0.734) **	0.243 (0.092, 0.394) **	−0.096(−0.303, 0.112)	−0.024 (−0.154, 0.106)	**0.012**
9 month	0.335 (0.065, 0.606) *	0.215 (0.062, 0.367) **	−0.048 (−0.257, 0.162)	−0.039 (−0.171, 0.093)	**0.037**
12 month	0.428 (0.166, 0.690) **	0.239 (0.093, 0.386) **	0.084 (−0.121, 0.290)	−0.042 (−0.171, 0.087)	**0.041**
18 month	0.340 (0.035, 0.645) *	0.145 (−0.024, 0.315)	−0.030 (−0.296, 0.235)	0.027 (−0.122, 0.177)	0.453
24 month	0.439 (0.146, 0.733) **	0.248 (0.086, 0.411) **	−0.037 (−0.292, 0.218)	−0.055 (−0.200, 0.091)	**0.025**
WAZ					
0 month	1.369 (1.156, 1.582) ***	0.303 (0.181, 0.425) ***	0.681 (0.512, 0.850) ***	0.039 (−0.064, 0.143)	**<0.001**
1 month	0.838 (0.584, 1.091) ***	0.206 (0.064, 0.349) **	0.306 (0.093, 0.518) **	−0.047 (−0.173, 0.079)	0.070
2 month	0.806 (0.585, 1.028) ***	0.206 (0.079, 0.332) **	0.335 (0.162, 0.509) ***	−0.011 (−0.122, 0.100)	**0.027**
4 month	0.815 (0.565, 1.066) ***	0.258 (0.116, 0.400) ***	0.394 (0.186, 0.602) ***	−0.036 (−0.159, 0.088)	**0.005**
6 month	0.718 (0.472, 0.965) ***	0.280 (0.140, 0.421) ***	0.326 (0.133, 0.519) **	−0.068 (−0.189, 0.053)	**0.001**
9 month	0.910 (0.667, 1.152) ***	0.239 (0.101, 0.377) ***	0.419 (0.230, 0.609) ***	0.069 (−0.050, 0.187)	0.057
12 month	0.865 (0.638, 1.093) ***	0.136 (0.007, 0.264) *	0.402 (0.222, 0.581) ***	0.138 (0.026, 0.251) *	0.095
18 month	0.817 (0.547, 1.087) ***	0.383 (0.233, 0.534) ***	0.276 (0.041, 0.512) *	0.113 (−0.019, 0.245)	0.135
24 month	0.992 (0.722, 1.262) ***	0.217 (0.066, 0.367) **	0.483 (0.248, 0.719) ***	0.232 (0.098, 0.365) **	0.228
LAZ					
0 month	0.313 (0.202, 0.424) ***	0.140 (0.052, 0.227) **	0.057 (−0.006, 0.119)	−0.033 (−0.086, 0.021)	**0.034**
1 month	0.646 (0.343, 0.949) ***	0.339 (0.170, 0.509) ***	0.221 (−0.032, 0.473)	−0.245 (−0.394, −0.095)	**<0.001**
2 month	0.565 (0.301, 0.830) ***	0.293 (0.143, 0.443) ***	0.132 (−0.074, 0.337)	−0.086 (−0.217, 0.046)	**0.006**
4 month	0.626 (0.364, 0.889) ***	0.316 (0.168, 0.465) ***	0.279 (0.062, 0.496) *	−0.135 (−0.272, 0.002)	**<0.001**
6 month	0.670 (0.414, 0.926) ***	0.318 (0.172, 0.464) ***	0.146 (−0.054, 0.346)	0.030 (−0.095, 0.156)	**0.040**
9 month	0.819 (0.555, 1.083) ***	0.201 (0.051, 0.351) **	0.248 (0.043, 0.454) *	0.068 (−0.061, 0.197)	0.548
12 month	0.844 (0.580, 1.108) ***	0.244 (0.095, 0.392) **	0.316 (0.108, 0.524) **	0.186 (0.056, 0.316) **	0.930
18 month	0.813 (0.493, 1.133) ***	0.224 (0.045, 0.403) *	0.256 (−0.023, 0.536)	0.242 (0.086, 0.399) **	0.707
24 month	0.594 (0.294, 0.895) ***	0.138 (−0.028, 0.304)	0.207 (−0.053, 0.468)	0.174 (0.025, 0.323) *	0.746
WHZ					
0 month	1.756 (1.457, 2.054) ***	0.415 (0.245, 0.585) ***	0.892 (0.656, 1.128) ***	0.097 (−0.048, 0.242)	**<0.001**
1 month	0.428 (0.128, 0.727) **	−0.134 (−0.302, 0.033)	0.246 (0.098, 0.394) **	0.243 (0.096, 0.391)	**0.002**
2 month	0.536 (0.278, 0.794) ***	−0.031 (−0.177, 0.116)	0.358 (0.157, 0.558) **	0.087 (−0.041, 0.216)	**0.017**
4 month	0.568 (0.303, 0.834) ***	0.079 (−0.072, 0.230)	0.310 (0.089, 0.531) **	0.065 (−0.066, 0.196)	0.314
6 month	0.458 (0.201, 0.716) ***	0.141 (−0.006, 0.288)	0.324 (0.123, 0.525) **	−0.114 (−0.240, 0.012)	**0.001**
9 month	0.677 (0.428, 0.926) ***	0.181 (0.039, 0.322) *	0.391 (0.198, 0.584) ***	0.046 (−0.075, 0.167)	0.057
12 month	0.663 (0.431, 0.894) ***	0.035 (−0.095, 0.166)	0.356 (0.173, 0.539) ***	0.079 (−0.036, 0.193)	**0.029**
18 month	0.571 (0.299, 0.844) ***	0.201 (−0.035, 0.437)	0.373 (0.222, 0.524) ***	−0.006 (−0.139, 0.126)	**0.004**
24 month	0.945 (0.661, 1.229) ***	0.187 (0.029, 0.345) *	0.542 (0.295, 0.789) ***	0.192 (0.050, 0.333) **	0.083

Adjusted for age at delivery, maternal pre-pregnancy BMI, education, income, physical activity, sleep quality, complications, parity, delivery mode, anxiety and depression in pregnancy, maternal passive smoking during pregnancy, maternal alcohol consumption before pregnancy, children’s sex, paternal age, paternal BMI, children’s age at anthropometric measurements, feeding mode, supplementary food addition. ^a^ Further adjusted for 1st-trimester GWGR. ^b^ Further adjusted for 1st-trimester GWGR and 2nd-trimester GWGR. ^c^ Further adjusted for energy intake in late pregnancy. ^d^ Score test of homogeneity of effect estimates across the three trimesters. When analyzing the physical growth of children during postnatal follow-up, we adjusted for SGA, LGA, and AGA. * p < 0.05, ** *p* < 0.01, *** *p* < 0.001.

**Table 3:** The effects of total and trimester-specific GWGR on children’s anthropometric outcomes from 0 to 24 months by linear mixed effects models (β (95% CI)) (n = 3229).

Children Anthropometric Measures	Total GWGR ^c^	1st Trimester GWGR	2nd Trimester GWGR ^a^	3rd Trimester GWGR ^bc^
	(kg/week)	(kg/week)	(kg/week)	(kg/week)
BMIZ	0.659 (0.483, 0.835) ***	0.133 (0.034, 0.233) **	0.312 (0.171, 0.452) ***	0.057 (−0.029, 0.143)
HCZ	0.422 (0.215, 0.629) ***	0.206 (0.090, 0.322) **	−0.017(−0.180−0.147)	−0.068 (−0.168, 0.033)
WAZ	0.854 (0.674, 1.034) ***	0.227 (0.125, 0.330) ***	0.339 (0.196, 0.482) ***	0.046 (−0.042, 0.134)
LAZ	0.642 (0.434, 0.849) ***	0.253 (0.136, 0.370) ***	0.192 (0.028, 0.356) *	−0.037 (−0.138, 0.064)
WHZ	0.626 (0.450, 0.802) ***	0.111 (0.012, 0.211) *	0.319 (0.178, 0.459) ***	0.091 (0.005, 0.176) *

Adjusted for age at delivery, maternal pre-pregnancy BMI, education, income, physical activity, sleep quality, complications, parity, delivery mode, anxiety and depression in pregnancy, maternal passive smoking during pregnancy, maternal alcohol consumption before pregnancy, children’s sex, paternal age, paternal BMI, breastfeeding duration, supplementary food addition. ^a^ Further adjusted for 1st-trimester GWGR. ^b^ Further adjusted for 1st-trimester GWGR and 2nd-trimester GWGR. ^c^ Further adjusted for energy intake in late pregnancy. * *p* < 0.05, ** *p* < 0.01, *** *p* < 0.001.

## Data Availability

The datasets presented in this article will be available for investigators after approval by Fudan University. Requests to access the datasets should be directed to Huijing Shi, hjshi@fudan.edu.cn.

## Supplementary Materials

The following supporting information can be downloaded at: https://www.mdpi.com/article/10.3390/nu15214523/s1, Table S1. Characteristics of participants during the period of the enrollment in Shanghai Maternal-Child Pairs Cohort (n = 6714), Table S2. Characteristics of the included and excluded populations (Mean ± SD or n (%)), Table S3: The effects of total and trimester-specific GWGR on children’s anthropometric outcomes from 0 to 24 months by linear mixed effects models [β (95% CI)] (n = 2884).
