# Supplementary material for: Prospective Association between Total and Trimester-Specific Gestational Weight Gain Rate and Physical Growth Status in Children within 24 Months after Birth
Source: Nutrients. 2023 Oct 25;15(21):4523. doi: 10.3390/nu15214523 (PMC10649666; doi:10.3390/nu15214523)
Supplement: Supplementary file 1 [file nutrients-15-04523-s001.zip › Table S1.pdf]

Table S1. Characteristics of participants during the period of the enrollment in Shanghai Maternal-Child Pairs Cohort (n=6714).

Characteristics	Mean ± SD or n (%)
**Maternal characteristics**	
District	
Pu dong	2963 (44.13)
Songjiang	3751 (55.87)
Age (ages)	28.89 ± 4.17
Education level	
Middle school and below	794 (11.83)
High school or same level	934 (13.91)
Junior college or same level	2213 (32.96)
College and above	2773 (41.30)
Race	
The Han nationality	6610 (98.45)
Other	104 (1.55)
Annual family income	
< ¥100,000	1794 (26.71)
¥100,000-200,000	3066 (45.67)
¥200,000-300,000	1217 (18.13)
⩾ ¥300,000	637 (9.49)
Pre-pregnancy BMI (kg/m^2)	
< 18.5	1081 (16.10)
18.5-24	4525 (67.40)
⩾ 24	1108 (16.50)
**Paternal characteristics**	
Age (years)	29.95 ± 4.71
FBMI category (kg/m^2)	
< 18.5	291 (4.33)
18.5-24	3403 (50.69)
⩾ 24	3020 (44.98)
