# Supplementary material for: Prospective Association between Total and Trimester-Specific Gestational Weight Gain Rate and Physical Growth Status in Children within 24 Months after Birth
Source: Nutrients. 2023 Oct 25;15(21):4523. doi: 10.3390/nu15214523 (PMC10649666; doi:10.3390/nu15214523)
Supplement: Supplementary file 1 [file nutrients-15-04523-s001.zip › Table S2.pdf]

Table S2. Characteristics of the included and excluded populations [Mean ± SD or n (%)].

Variables	Subjects included in this analyses (n=3229)	Subjects excluded in this analyses (n=1714)	*P*-Value
**Maternal characteristics**			
Age at delivery (years)	28.93 ± 4.09	28.72 ± 4.06	0.871
Education level			0.361
Middle school and below	285 (8.82)	150 (8.76)	
High school or same level	410 (12.70)	223 (13.00)	
Junior college or same level	1081 (33.48)	592 (34.56)	
College and above	1453 (45.00)	749 (43.68)	
Annual family income			0.386
< ¥100,000	771 (23.88)	416 (24.28)	
¥100,000-200,000	1551 (48.03)	828 (48.32)	
¥200,000-300,000	600 (18.58)	315 (18.39)	
⩾ ¥300,000	307 (9.51)	154 (9.01)	
Second-hand smoking during pregnancy			0.337
Yes	781 (24.19)	401 (23.40)	
No	2448 (75.81)	1277 (74.51)	
Alcohol before pregnancy	74 (2.29)	36 (2.09)	
Sleep quality during pregnancy			0.362
Good	1099 (34.04)	611 (35.62)	
Poor	2130 (65.96)	1103 (64.38)	
Physical activity intensity			0.154
Low	1327 (41.10)	697 (40.65)	
Medium	1743 (53.98)	924 (53.93)	
High	159 (4.92)	93 (5.42)	
Depression during pregnancy	366 (11.33)	187 (10.91)	0.578
Anxiety during pregnancy	401 (12.42)	225 (13.15)	0.611
Parity			0.123
Primiparous	1803 (55.84)	960 (56.01)	
Non-primiparous	1426 (44.16)	754 (43.99)	
Pre-pregnancy BMI (kg/m^2)			0.412
< 18.5	543 (16.82)	293 (17.12)	
18.5-24	2170 (67.20)	1164 (67.89)	
⩾ 24	516 (15.98)	257 (14.99)	
Gestational Diabetes	345(10.68)	172 (10.01)	0.144
**Paternal characteristics**			
Age (years)	29.97 ± 4.62	29.63 ± 4.53	0.779
FBMI category (kg/m^2)			0.414
< 18.5	140 (4.34)	71 (4.12)	
18.5-24	1615 (50.02)	860 (50.17)	
⩾ 24	1474 (45.64)	783 (45.71)	

**Children's characteristics**			
Sex			0.376
Male	1651 (51.13)	892 (52.04)	
Female	1578 (48.87)	882 (47.96)	
