# Supplementary material for: Prospective Association between Total and Trimester-Specific Gestational Weight Gain Rate and Physical Growth Status in Children within 24 Months after Birth
Source: Nutrients. 2023 Oct 25;15(21):4523. doi: 10.3390/nu15214523 (PMC10649666; doi:10.3390/nu15214523)
Supplement: Supplementary file 1 [file nutrients-15-04523-s001.zip › Table S3.pdf]

Table S3. The effects of total and trimester-specific GWGR on children's anthropometric outcomes from 0 to 24 months by linear mixed effects models [β (95% CI)] (n = 2884).

**Children Anthropometric Measures**	**Total GWGR** [c] (kg/week)	**1st Trimester GWGR** (kg/week)	**2nd Trimester GWGR** [a] (kg/week)	**3rd Trimester GWGR** [bc] (kg/week)
BMIZ	0.642 (0.453, 0.831)***	0.141 (0.037, 0.246)**	0.337 (0.180, 0.494)***	0.068 (-0.026, 0.162)
HCZ	0.458 (0.236, 0.680)***	0.231 (0.109, 0.353)***	-0.032 (-0.215, 0.152)	-0.035 (-0.144, 0.074)
WAZ	0.833 (0.637, 1.024)***	0.219 (0.111, 0.326)***	0.375 (0.214, 0.535)***	0.050 (-0.046, 0.146)
LAZ	0.620 (0.397, 0.843)***	0.231 (0.108, 0.354)***	0.206 (0.022, 0.391)*	-0.036 (-0.147, 0.074)
WHZ	0.617 (0.428, 0.806)***	0.116 (0.011, 0.220)*	0.345 (0.188, 0.501)***	0.108 (0.015, 0.202)*

Adjusted for age at delivery, maternal pre-pregnancy BMI, education, income, physical activity, sleep quality, complications, parity, delivery mode, anxiety and depression in pregnancy, maternal passive smoking during pregnancy, maternal drinking alcohol before pregnancy, children's sex, paternal age, paternal BMI, breastfeeding duration, supplementary food addition. [a] Further adjusted for 1st Trimester GWGR. [b] Further adjusted for 1st Trimester GWGR and 2nd Trimester GWGR. [c] Further adjusted for energy intake in late pregnancy. * $P < 0.05$, ** $P < 0.01$, *** $P < 0.001$.
